# The Multidimensional Prognostic Index predicts incident delirium among hospitalized older patients with COVID-19: a multicenter prospective European study

**DOI:** 10.1007/s41999-024-00987-y

**Published:** 2024-06-15

**Authors:** Wanda Morganti, Carlo Custodero, Nicola Veronese, Eva Topinkova, Helena Michalkova, M. Cristina Polidori, Alfonso J. Cruz‐Jentoft, Christine A. F. von Arnim, Margherita Azzini, Heidi Gruner, Alberto Castagna, Giovanni Cenderello, Romina Custureri, Emanuele Seminerio, Tania Zieschang, Alessandro Padovani, Elisabet Sanchez‐Garcia, Alberto Pilotto, the MPI-COVID-19 Study Group Investigators, Mario Barbagallo, Marina Barbagelata, Simone Dini, Naima Madlen Diesner, Marilia Fernandes, Federica Gandolfo, Sara Garaboldi, Clarissa Musacchio, Andrea Pilotto, Lena Pickert, Silvia Podestà, Giovanni Ruotolo, Katiuscia Sciolè, Julia Schlotmann

**Affiliations:** 1 Department of Geriatric Care, Neurology and Rehabilitation, Galliera Hospital, Genoa, Italy; 2 Department of Interdisciplinary Medicine, “Aldo Moro” University of Bari, Bari, Italy; 3 Department of Internal Medicine and Geriatrics, University of Palermo, Palermo, Italy; 4 Department of Geriatrics, First Faculty of Medicine, Charles University, Prague, Czech Republic; 5 Faculty of Health and Social Sciences, University of South Bohemia, Ceske Budejovice, Czech Republic; 6 Department II of Internal Medicine and Center for Molecular Medicine Cologne, Faculty of Medicine, Ageing Clinical Research, University Hospital Cologne, Cologne, Germany; 7 Cologne Excellence Cluster on Cellular Stress Responses in Aging- Associated Diseases (CECAD), University of Cologne, Cologne, Germany; 8 Servicio de Geriatría, Hospital Universitario Ramón y Cajal (IRYCIS), Madrid, Spain; 9 Department of Geriatrics, University Medical Center Göttingen, Göttingen, Germany; 10 Geriatrics Unit, “Mater Salutis” Hospital, Legnago ULSS 9 Scaligera, Verona, Italy; 11 Serviço de Medicina Interna, Hospital Curry Cabral, Centro Hospitalar Universitário Lisboa Central/Universidade Nova de Lisboa, Lisbon, Portugal; 12 Geriatrics Unit, “Pugliese Ciaccio” Hospital, Catanzaro, Italy; 13 Infectious Disease Unit, Sanremo Hospital, ASL 1 Imperiese, Sanremo, Italy; 14 University-Clinic for Geriatric Medicine, Klinikum Oldenburg AöR, Oldenburg University, Oldenburg, Germany; 15 Neurology Unit, Department of Clinical and Experimental Sciences, University of Brescia, Brescia, Italy

**Keywords:** Multidimensional Prognostic Index, Delirium prediction, Comprehensive geriatric assessment, COVID-19, Older people

## Abstract

**Purpose:**

Incident delirium is a frequent complication among hospitalized older people with COVID-19, associated with increased length of hospital stay, higher morbidity and mortality rates. Although delirium is preventable with early detection, systematic assessment methods and predictive models are not universally defined, thus delirium is often underrated. In this study, we tested the role of the Multidimensional Prognostic Index (MPI), a prognostic tool based on Comprehensive Geriatric Assessment, to predict the risk of incident delirium.

**Methods:**

Hospitalized older patients (≥ 65 years) with COVID-19 infection were enrolled (n = 502) from ten centers across Europe. At hospital admission, the MPI was administered to all the patients and two already validated delirium prediction models were computed (AWOL delirium risk-stratification score and Martinez model). Delirium occurrence during hospitalization was ascertained using the 4A’s Test (4AT). Accuracy of the MPI and the other delirium predictive models was assessed through logistic regression models and the area under the curve (AUC).

**Results:**

We analyzed 293 patients without delirium at hospital admission. Of them 33 (11.3%) developed delirium during hospitalization. Higher MPI score at admission (higher multidimensional frailty) was associated with higher risk of incident delirium also adjusting for the other delirium predictive models and COVID-19 severity (OR = 12.72, 95% CI = 2.11–76.86 for MPI-2 vs MPI-1, and OR = 33.44, 95% CI = 4.55–146.61 for MPI-3 vs MPI-1). The MPI showed good accuracy in predicting incident delirium (AUC = 0.71) also superior to AWOL tool, (AUC = 0.63) and Martinez model (AUC = 0.61) (*p* < 0.0001 for both comparisons).

**Conclusions:**

The MPI is a sensitive tool for early identification of older patients with incident delirium.

**Supplementary Information:**

The online version contains supplementary material available at 10.1007/s41999-024-00987-y.

## Key summary points

**Aim:**

Testing the role of the Multidimensional Prognostic Index (MPI), based on the Comprehensive Geriatric Assessment (CGA), in predicting the risk of incident delirium in hospitalized older patients with COVID-19.

**Findings:**

The MPI showed a good accuracy in predicting incident delirium (AUC = 0.71). Its accuracy is higher than the ones of two validated predictive models (AWOL delirium risk-stratification score’s AUC = 0.63; Martinez Model’s AUC = 0.61; p < 0.0001 for both comparisons).

**Message:**

The MPI is a sensitive tool for risk-stratification of the incident delirium in hospitalized older COVID-19 patients.

**Supplementary Information:**

The online version contains supplementary material available at 10.1007/s41999-024-00987-y.

## Introduction

Delirium, as defined by the DSM-5 [1] criteria, consists of a disturbance in attention and awareness, together with a cognitive change, developed over hours or a few days and representing a severe change from baseline functioning. It is a neuropsychiatric syndrome common among older people and is the most frequent hospital admission complication, [2] with an occurrence ranging from 11 to 42% [3] of patients, especially after surgery [4].

As opposed to prevalent delirium which stands for the insurgence of delirium at admission in the Emergency Department (ED), incident delirium can be applied to patients who were non-delirious at hospital admission and develop delirium during hospitalization or ED stay [5]. Delirium etiology is quite heterogeneous, involving some predisposing factors, such as old age, sensory impairments, presence of severe illnesses and cognitive impairment, and triggering or precipitating factors such as dehydration [6], infection, malnutrition, polypharmacy, environmental changes, and, especially for incident delirium, the occurrence of iatrogenic events [2, 7]. Incident delirium occurring in ED is associated with higher morbidity and mortality risks together with an increase in-hospital stay (21 days Vs. 9 days without delirium), and a greater risk of developing dementia and loss of independence [7].

However, because delirium could be prevented with tailored interventions [8–11] early identification of patients at risk for delirium seems to be important. In spite of the availability in clinical practice of several tools for the early delirium risk assessment, a systematic method is not universally defined and delirium is often underrated [12]. Furthermore, predictive models are quite heterogeneous, focusing on different risk factors and addressing diverse populations [2].

A recent review identified several delirium prediction models [13] with varying degrees of accuracy (area under the curve-AUC from 0.52 to 0.94). Easy-to-assess but reliable prediction models for the assessment at hospital admission are the AWOL delirium risk-stratification score [14] and the Martinez model [6], both including age and then focusing on cognition, disorientation, and illness severity for the former, and dependence and dementia diagnosis for the latter. Moreover, a systematic review with meta-analysis [15] highlighted a 2.2-fold greater risk of developing delirium in frail individuals, stressing the usefulness of deepening the knowledge about the possible relation between these two conditions and the role of frailty as a predisposing factor for delirium, as it can multidimensionally contribute to susceptibility to negative outcomes [15]. In addition, delirium could be the phenotypic presentation and the neuropsychiatric manifestation of an underlying frailty condition. This has emerged with particular strength during the COVID-19 pandemic, in which delirium often represented an atypical presentation of the disease [16] in frail older adults.

The Multidimensional Prognostic Index (MPI) is a prognostic tool derived from the Comprehensive Geriatric Assessment (CGA) which is able to stratify older adults based on the risk of negative outcomes [17, 18] and may help in daily practice for the clinical decision-making [19]. Recently, the MPI demonstrated to accurately predict pre-operative delirium in older adults undergoing surgery for hip fracture [20]. However, no studies evaluated the potential predictive value of the MPI in general medicine wards to identify the subjects at higher risk of delirium during hospitalization.

Given this background and the inconsistency of the currently available delirium predictive models, along with the importance of reducing assessment time while remaining reliable and precise, we tested the ability of the MPI to predict the risk of incident delirium among older adults hospitalized with COVID-19 disease.

## Materials and methods

### Study population

This study is a longitudinal observational cohort study that was carried out in compliance with the Declaration of Helsinki and formally authorized by the local ethical committees of each participating institution. Participants were older subjects consecutively admitted to the hospital with a diagnosis of COVID-19 infection, enrolled from April 2020 to August 2021. Patients were hospitalized in general medicine wards (i.e., geriatrics, internal medicine units) from 10 European centers located in Italy (5 centers, 272 participants), Spain (1 center, 46 participants), Czech Republic (1 center, 153 participants), Portugal (1 center, 34 participants), and Germany (2 centers, 43 participants). Inclusion criteria were a) being at least 65 years old, b) consecutively being admitted to the hospital with a COVID-19 diagnosis made through a nasopharyngeal swab, and c) willingness to participate in the study. Exclusion criteria were age under 65 years and being unwilling or unable to provide informed consent.

Informed consent was given by the participants for their clinical records to be used in clinical studies: since the patients could be not able to understand the aims of the study (e.g., for severe hypoxemia), we recorded informed consent until 48 h after the admission. COVID-19 severity was defined as the use of non-invasive ventilation (NIV) or oro-tracheal intubation during the hospitalization. All the patient records were anonymized and de-identified before the analyses.

### Exposure: the Multidimensional Prognostic Index (MPI)

The MPI [17] is a widely used and validated CGA-based [19] instrument for the assessment of multidimensional frailty in hospitalized older people, able to predict negative outcomes (e.g., rehospitalization, institutionalization, mortality and falls) [18]. This tool has been already demonstrated to be feasible also in patients with respiratory failure and hospitalized with COVID-19 disease [21, 22]. The MPI explored functional, nutritional, cognitive and social status, levels of mobility, comorbidities and polypharmacy (see Supplementary Materials).

The final score range between 0 = no risk and 1 = higher risk of mortality and can be classified as MPI-1 (low risk of frailty, MPI index under 0.33), MPI-2 (moderate risk of frailty, MPI index between 0.34 and 0.66), or MPI-3 (high risk of frailty, MPI index greater than 0.67). The MPI was administered during the first 24–48 h from the admission by a health-professional.

### Delirium assessment: the 4 “A”s test (4AT)

For delirium detection, we used the 4AT, a simple and reliable instrument. The administration takes about 2 min and does not require any training. Furthermore, vision or hearing impairment does not interfere with the examination. It is composed of four items: 1. level of Alertness [23]; 2. a brief cognitive assessment through the Abbreviated Mental Test-4 [24]; 3. Attention evaluation [25]; 4. Acute change or fluctuating mental status occurring within the last 2 weeks and enduring in the last 24 h [26]. Each item was summed to obtain a score from 0 to 12, with 4 as a cut-off for possible delirium [27]. For delirium, the 4AT’s sensitivity is 89.7% and its specificity is 84.1% [27]. In the study, the 4As Test was routinely administered at admission and on discharge and also at any time during hospitalization when delirium is suspected based on clinical observation.

### Delirium prediction models

Based on a previous systematic review by Lindroth and colleagues we identified all the potential delirium prediction tools [13]. Given the retrospective nature of the analysis we selected those tools that could be calculated from the available information in our dataset. Thus, as the study’s delirium prediction tools, the AWOL delirium risk-stratification score and the Martinez model (as modified by [28]) were used:

- **The AWOL delirium risk-stratification score** is calculated by giving 1 point each to increased nurse-rated illness severity and age over 80 years and 2 points to dementia diagnosis and/or Mini Mental State Examination (MMSE) [29] score < 24, or Abbreviated Mental Test Score (AMTS) [30] score < 9;
- **The Martinez model** predicts delirium based on the presence of three criteria: age over 85 years, loss of independence in at least five ADLs, and cognitive impairment based on MMSE (score < 24) or AMTS (score < 9).

In the present study, we used as cognitive rating an SPMSQ score higher or equal to 8 according to the previously validated comparison with an MMSE score < 24 [31, 32].

## Statistical analysis

The descriptive characteristics of the study population were expressed as means and standard deviations for continuous variables and percentages (%) for categorical variables. The Shapiro–Wilk test was used to determine the normality of distributions. For the comparison of continuous variables between subjects with and without incident delirium, independent sample *t*-tests (or the equivalent nonparametric test) were used. The percentages of the categorical variables were compared using Chi-square tests for the same two subsamples. To test the associations between the diagnosis of incident delirium and MPI (adjusting for age and gender and for age, gender, AWOL delirium risk-stratification score, Martinez model and COVID-19 severity), logistic regression models were used to calculate odds ratios (ORs) and their 95% confidence intervals (CIs).

Finally, the AUC was examined to gage how well the MPI, the AWOL delirium risk-stratification score, and the Martinez model predicted the diagnosis of incident delirium. The AUCs were compared using the test proposed by DeLong et al. [33]. All analyses were conducted using SPSS (Version 26.0) and all two-tailed statistical tests were deemed statistically significant at a *p*-value of 0.05 or less.

## Results

From the initial study population of 502 patients, 206 (41%) were excluded having delirium at admission (4AT score ≥ 4/12). Moreover, three participants had missing data and were also excluded from the analysis. Therefore, the final sample was composed of 293 patients (57% females), of which 33 (11.3%) developed incident delirium during their hospitalization.

Table 1 shows the baseline characteristics of the sample classified according to the development of delirium during the hospitalization. Patients who had delirium were older than the ones who did not develop it (82.7 ± 7.3 vs. 79.2 ± 8.1, *p* = 0.018). Blood parameters (including pO2) and clinical and immunologic status did not differ between patients with and without delirium. The scores of the delirium predictive models were significantly higher (higher delirium risk) among subjects who developed delirium (AWOL delirium risk-stratification score: 1.394 ± 0.899 vs. 0.850 ± 0.817, *p* < 0.001; Martinez model: 1.333 ± 0.889 vs. 0.977 ± 0.795; *p* = 0.002). Moreover, the MPI score in the delirium group was higher than the comparison group without delirium (0.59 ± 0.19 vs. 0.43 ± 0.22; *p* < 0.001), with significantly poorer scores in the following MPI-domains: IADL, cognitive status, mobility, and number of medications.

**Table 1:** Baseline descriptive characteristics, by incident delirium during the follow-up

Parameter	All sample		
	Delirium (*n *= 33)	No delirium (*n* = 260)	*p*-value
Mean age (mean, SD)	82.7 (7.3)	79.2 (8.1)	0.018*
Female gender (%)	48.5	58.1	0.294
MPI domains (means, SDs)			
ADL score	3.7 (2.0)	4.2 (1.9)	0.185
IADL score	3.0 (2.4)	4.8 (2.4)	0.001*
SPMSQ score	4.6 (2.5)	3.4 (2.2)	0.006*
ESS score	14.4 (4.0)	16.1 (3.2)	0.002*
MNA-SF score	8.1 (3.3)	9.2 (3.3)	0.061
CIRS-CI score	4.3 (1.8)	3.7 (2.1)	0.101
Number of medications	7.4 (3.3)	5.9 (3.1)	0.012*
Living alone (%)	9.1	23.9	0.267
MPI score	0.59 (0.19)	0.43 (0.22)	< 0.0001*
Blood parameters (means, SDs)			
CRP	16.7 (23.8)	10.8 (16.2)	0.188
PO_2_	57.3 (18.2)	45.3 (25.8)	0.330
SatO_2_	85.3 (15.9)	90.9 (11.5)	0.081
Clinical and immunologic status (%)			
Dyspnea	42.4	57.5	0.100
Cough	42.4	43.0	0.948
Fever	48.5	50.6	0.821
Diarrhea	24.2	15.1	0.180
Clinical scores predicting delirium			
AWOL delirium risk-stratification score	1.394 (0.899)	0.850 (0.817)	< 0.0001*
Martinez model	1.333 (0.889)	0.977 (0.795)	0.002*

*SD* standard deviation*; MPI* Multidimensional Prognostic Index; *ADL* activities of daily living*; IADL* instrumental activities of daily living*; SPMSQ* short portable mental status questionnaire*; ESS* exton smith scale; *MNA-SF* mini-nutritional assessment, short form; *CIRS-CI* cumulative illness rating scale-comorbidity index*; CRP* c-reactive protein; *PO*_2_ partial pressure of oxygen*; SatO*_2_oxygen saturation of arterial blood

*Statistically significant *p*-value

Compared to subjects in the low-risk category (MPI-1) at hospital admission, those in the moderate-risk (MPI-2) as well as those in the high-risk MPI category (MPI-3) showed higher risk of developing incident delirium independently of age, gender, AWOL delirium risk-stratification score and Martinez model and COVID-19 severity (OR = 12.72, 95% CI = 2.11–76.86, *p* = 0.006 for MPI-2 vs. MPI-1; OR = 33.44, 95% CI = 4.55–146.61, *p* = 0.001 for MPI-3 vs. MPI-1) (Table 2).

**Table 2:** Logistic regression model for the prediction of incident delirium

MPI categories	Model 1			Model 2		
	OR	95% CI	*p*-value	OR	95% CI	*p*-value
MPI-1	Reference			Reference		
MPI-2	5.353	1.107–25.886	0.037	12.72	2.11–76.86	0.006
MPI-3	11.087	2.242–54.825	0.003	33.44	4.55–146.61	0.001

All data are reported as odds ratios (ORs) with their 95% confidence intervals (CIs). Model 1 was adjusted for age and gender, model 2 was adjusted for age, gender, AWOL delirium risk-stratification score and Martinez model and COVID-19 severity

**Fig. 1:**
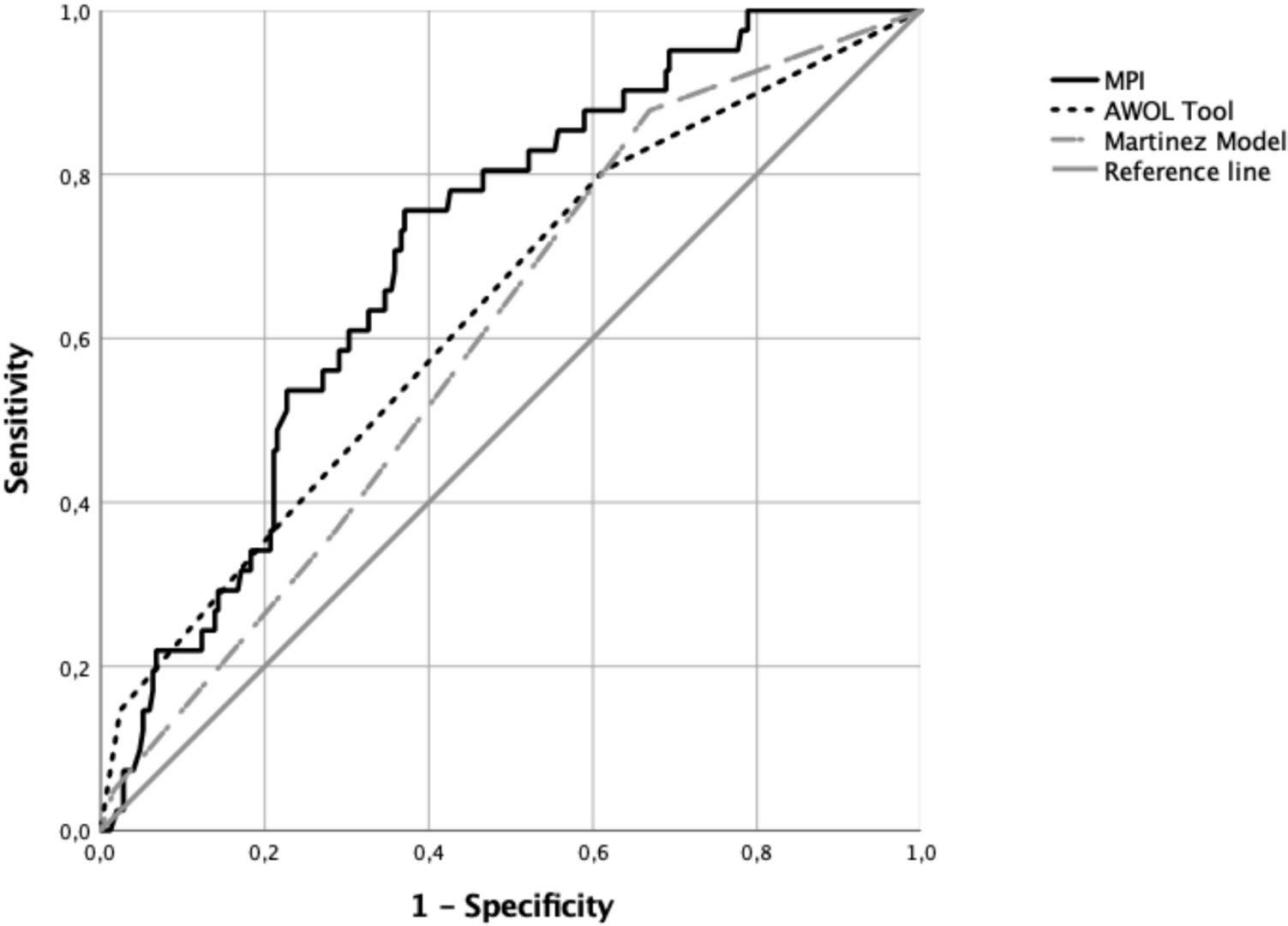
Accuracy of Multidimensional Prognostic Index (MPI), AWOL delirium risk-stratification score and Martinez models in predicting incident delirium

We calculated the AUC of the MPI and the two previously validated predictive models (the AWOL delirium risk-stratification score and the Martinez model) to test MPI’s accuracy in predicting incident delirium. As shown in Fig. 1 and Table 3, the MPI’s Receiver Operating Characteristic (ROC) Curve Area was 0.71 (*p* < 0.001), indicating that the MPI can predict nearly 71% of subjects with incident delirium and indicating that MPI was more accurate in predicting incident delirium compared to the AWOL delirium risk-stratification score (AUC = 0.63) and the Martinez model (AUC = 0.61), respectively (*p* < 0.0001 for both comparisons). An MPI absolute value of 0.33 shows a sensitivity of 95% and a specificity of 25% while a score of 0.66 has a sensitivity of 59% and a specificity of 70%.

**Table 3:** Area under the curve and confidence intervals of the MPI and of the two other predictive models based on the capacity to predict incident delirium

Parameter	Area	Standard error	*p*-value	95% Confidence intervals	
				Lower	Higher
MPI	0.707	0.039	< 0.0001	0.631	0.782
AWOL delirium risk-stratification score	0.634	0.047	0.006	0.541	0.727
Martinez model	0.605	0.044	0.031	0.519	0.691

## Discussion

In this study, we found that a CGA-based prognostic instrument, such as the MPI, performed at hospital admission can be an accurate tool for predicting delirium risk during hospitalization in older adults with COVID-19. The MPI identified with good accuracy patients at risk for delirium (AUC = 0.71) and showed greater discriminatory power compared to some currently adopted delirium prediction tools, i.e., the AWOL delirium risk-stratification score and the Martinez model.

Delirium is frequently reported as concomitant to SARS-CoV-2 infection, developing roughly in one out of five older subjects hospitalized with COVID-19 [34]. Compared to the pre-pandemic period when delirium prevalence was reported as higher, the estimates across the COVID-19 waves showed a tendency to decrease probably due to the vaccination programs which attenuated the COVID-19 severity [35, 36]. Occurrence of delirium is higher among frail subjects compared to non-frail reaching a prevalence of 37%.

The study was conducted until the third pandemic wave, on a sample of hospitalized older adults with COVID-19 which showed a 41% prevalence of delirium, similar to previously reported estimates, [37] and a slightly lower incidence (about 11%) probably because the delirium was more frequently identified in the emergency department.

Delirium may represent a sentinel event, predisposing to a higher risk of morbidity and mortality [16]. Instruments able to recognize older subjects at risk for delirium are strategic to start early preventive interventions. A recent systematic review summarized evidence on 14 externally validated delirium prediction models [13]. The items more often included in these instruments were cognitive impairment, sensory deficit, advanced age, poor functional status, illness severity, history of alcohol consumption, and presence of infectious disease. Collectively, such tools showed variable and, in most cases, inadequate predictive capabilities [13]. Moreover, very few instruments have been specifically developed for general medicine settings and often showed a high risk of bias and poor generalizability [38, 39]. Some delirium prediction models have also been specifically developed and validated for COVID-19 disease. For example, Castro et al. proposed an electronic health records-based tool built upon a machine-learning approach derived from demographic, clinical, laboratory, and medication information. This model showed an AUC of 0.75 for incident delirium, but the accuracy decreased in older adults (AUC = 0.67) and those with a history of dementia (AUC = 0.58) [40]. Conversely, the MPI, used in our study conducted on an older population, showed better performance in predicting delirium risk (AUC = 0.71), compared to two already validated delirium prediction tools as well (AWOL and the Martinez model).

This might suggest that information routinely collected from a standard CGA could be able to identify older adults hospitalized for COVID-19 who are more prone to develop delirium. Moreover, we found that older in-patients who developed delirium during hospital stay were significantly older, took a higher number of medications, and had lower cognitive performance and functional status compared to those patients who did not have delirium. At admission, subjects who developed delirium showed higher levels of multidimensional impairment assessed by the MPI. Furthermore, higher MPI levels (MPI-2 and MPI-3) may predict a greater risk of developing delirium during the follow-up, compared to the lowest risk MPI category. Previous evidence already highlighted that the MPI can predict pre-operative delirium in older adults with hip fractures [20], emphasizing that a standardized CGA might allow the identification of older subjects at risk for delirium in very heterogeneous settings and independently by age, gender, and setting-specific risk factors. Such a strict association between multidimensional frailty and delirium might be explained also at a biologic level by common pathogenetic mechanisms such as the emerging role of systemic inflammation [41, 42].

Our study demonstrated that a multidimensional assessment using the MPI has greater accuracy in predicting the occurrence of delirium, compared to two other validated prediction models for delirium. Overall, our data corroborate the theory that multidimensional aggregate information, readily available in clinical practice and easy to obtain, could aid physicians in predicting mortality, as previously reported, and the occurrence of delirium as well.

We should acknowledge some limitations of this study. The retrospective nature of the analysis did not allow the collection of potentially relevant information such as delirium motor subtypes (hyperactive, hypoactive, mixed), delirium delay from admission, residual confounders (e.g., other well-known precipitating factors including medications, procedures, and use of devices), and different delirium prediction models. Furthermore, it is well recognized in the scientific literature that clinical judgment might indeed underestimate the adequate identification of delirium [43], thus the use of 4AT assessment prompted by clinical suspicion and not carried out daily could have underestimated the true incidence of delirium in this population. Moreover, follow-up information on delirium occurrence after the index hospitalization was not available. Finally, we did not collect detailed information about the medications used: therefore, we could not consider the role of this specific factor in determining incident delirium.

In conclusion, a CGA-based tool, such as the MPI, when performed at hospital admission, might represent a sensitive instrument predicting delirium in older adults with COVID-19 disease. This tool outperformed prediction models specifically validated for delirium, identifying subjects at risk who may need an individualized approach to prevent delirium occurrence. Future research should test the usefulness of personalized clinical approaches guided by this CGA-based tool in delirium prevention.

## Supplementary Information

Below is the link to the electronic supplementary material.

Supplementary file1 (DOCX 20 KB)

## Data Availability

The datasets generated and/or analysed during the current study are not publicly available. However, the datasets are available from the corresponding author on reasonable request.
