# Supplementary material for: The Multidimensional Prognostic Index predicts incident delirium among hospitalized older patients with COVID-19: a multicenter prospective European study
Source: Eur Geriatr Med. 2024 Jun 15;15(4):961–9. doi: 10.1007/s41999-024-00987-y (PMC11377617; doi:10.1007/s41999-024-00987-y)
Supplement: Supplementary file 1 — Supplementary file1 (DOCX 20 KB) [file 41999_2024_987_MOESM1_ESM.docx]

**Supplementary Material: the Multidimensional Prognostic Index**

It is composed of 51 items included in six domains assessed by eight scales:

- functional status and independence using the basic (ADL)[1] and instrumental activities of daily living (IADL)[2];
- mobility condition/risk of developing sore pressures through the Exton Smith Scale (ESS) [3]
- nutritional status with the Mini Nutritional Assessment – Short Form (MNA-SF) [4];
- cognitive status through the Short Portable Mental Status Questionnaire (SPMSQ) [5];
- health status namely comorbidity, using the Comorbidity Index of the Cumulative Illness Rating Scale (CIRS-CI) [6], and the number of drugs taken;
- the cohabitation status (living alone, with family or institutionalized).

Its administration takes 15 minutes on average [7].

Each scale outputs a risk score category (0=low risk; 0.5=mild risk; 1=severe risk), which is aggregated and then divided by the number of completed scales (at least 6).

**References**

1. Katz S, Downs TD, Cash HR, Grotz RC. Progress in Development of the Index of ADL. The Gerontologist 1970; 10:20–30.

2. Lawton MP, Brody EM. Assessment of Older People: Self-Maintaining and Instrumental Activities of Daily Living. The Gerontologist 1969; 9:179–186.

3. Bliss MR, McLaren R, Exton-Smith AN. Mattresses for preventing pressure sores in geriatric patients. Mon Bull Minist Health Public Health Lab Serv 1966; 25:238–268.

4. MNA-International Group, Kaiser MJ, Bauer JM, Ramsch C, Uter W, Guigoz Y, Cederholm T, Thomas DR, Anthony P, Charlton KE, Maggio M, Tsai AC, Grathwohl D, Vellas B, Sieber CC. Validation of the Mini Nutritional Assessment short-form (MNA®-SF): A practical tool for identification of nutritional status. J Nutr Health Aging 2009; 13:782–788.

5. Pfeiffer E. A Short Portable Mental Status Questionnaire for the Assessment of Organic Brain Deficit in Elderly Patients†. Journal of the American Geriatrics Society 1975; 23:433–441.

6. Linn BS, Linn MW, Gurel L. CUMULATIVE ILLNESS RATING SCALE. Journal of the American Geriatrics Society 1968; 16:622–626.

7. Bonnekoh SI, Meyer AM, Pickert L, Schulz R-J, Becker I, Polidori MC. The multidimensional prognostic index in hospitalized older adults: practicability with regard to time needs. Aging Clin Exp Res 2023; 35:711–716.
